# Development and Characterization of the Shortest Anti-Adhesion Peptide Analogue of B49Mod1

**DOI:** 10.3390/molecules25051188

**Published:** 2020-03-06

**Authors:** Yuan Lyu, Wadie D. Mahauad-Fernandez, Chioma M. Okeoma

**Affiliations:** 1 Department of Pharmacology, Stony Brook University Renaissance School of Medicine, Stony Brook, NY 11794-8651, USA; yuan.lyu@stonybrook.edu; 2 Division of Oncology, Departments of Medicine and Pathology, Stanford University School of Medicine, 291 Campus Drive, Stanford, CA 94305, USA; wmahauad@stanford.edu

**Keywords:** BST-2, anti-adhesion peptide, B49/B49Mod1, B18, breast cancer cells

## Abstract

Inhibition of cancer cell adhesion is an effective approach to killing adherent cancer cells. B49 and its analog B49Mod1 peptides, derived from the extracellular domain (ECD) of bone marrow stromal antigen 2 (BST-2), display anti-adhesion activity on breast cancer cells. However, the minimal sequence required for this anti-adhesion activity is unknown. Here, we further characterized the anti-adhesion activity of B49Mod1. We show that the anti-adhesion activity of B49Mod1 may require cysteine-linked disulfide bond and that the peptide is susceptible to proteolytic deactivation. Using structure-activity relationship studies, we identified an 18-Mer sequence (B18) as the minimal peptide sequence mediating the anti-adhesion activity of B49Mod1. Atomistic molecular dynamic (MD) simulations reveal that B18 forms a stable complex with the ECD of BST-2 in aqueous solution. MD simulations further reveal that B18 may cause membrane defects that facilitates peptide translocation across the bilayer. Placement of four B18 chains as a transmembrane bundle results in water channel formation, indicating that B18 may impair membrane integrity and form pores. We hereby identify B18 as the minimal peptide sequence required for the anti-adhesion activity of B49Mod1 and provide atomistic insight into the interaction of B18 with BST-2 and the cell membrane.

## 1. Introduction

Peptide-based therapeutics is an emergent field for anti-cancer agents with significant benefits that includes ease of production and flexibility for modification. As a result, efforts are being made to develop efficient drugs to treat cancer and control metastasis. Cancer metastasis is a complex process involving dissociation of tumor cells from the primary tumor, invasion of the tumor cells into the surrounding tissue, extravasation from circulation, and seeding/growth in distant organs. Tumor cells mimic leukocytes to enable transmigration through the endothelial barrier at the metastatic site. The attachment of leukocytes/cancer cells to the endothelium is mediated by several cell adhesion molecules (CAMs) different from those at the site of the primary tumor. Cancer cell adhesion is a biological process that is key to the metastatic cascade [1]. Expression of cell-to-cell and cell-to-matrix adhesion molecules, their regulation (up or down), and activation/deactivation have been linked to cancer cell migration and the metastatic cascade [2]. Thus, it is important to identify the modulators of cancer cell adhesion and to develop molecules that can inhibit cell adhesion.

One such novel cancer cell adhesion regulator is BST-2, a type II transmembrane protein of 180 amino acids [3]. BST-2 is known for its antiviral properties [4,5]. The unique topology of BST-2 and its ability to induce intracellular signaling enables it to retain virions on the cell surface and to block viral replication both in cells and in vivo [6,7,8]. BST-2 consists of an N-terminal intracellular domain (ICD), a transmembrane (TM) region, and a C-terminal glycosyl-phosphatidylinositol (GPI) anchor [9] which are separated by ∼120 residues constituting the extracellular domain (ECD) [10,11]. 

BST-2 ICD contains two tyrosine residues that mediate intracellular signal transduction, and its ECD contains three cysteine residues at positions 53, 63, and 91 that mediate cysteine-linked BST-2 dimerization [12,13,14]. During virus tethering, monomeric BST-2 is incorporated into viral membranes [15]. The virus-borne BST-2 dimerizes with cell-associated BST-2, thereby facilitating virus tethering and promoting cell-cell viral spread through the formation of viral clusters [16]. The BST-2-mediated inhibition of virus release and promotion of cell-cell virus spread is an example of the paradoxical role played by BST-2 in infected cells that is analogous to the role of BST-2 in regulating cancer cell adhesion. Findings from many labs have shown that BST-2 is overexpressed in several cancers including lung cancer [17], head and neck carcinomas [18], oral cavity cancers [19], glioblastomas [20], endometrial cancer [21], lymphomas [22], and breast cancer [23,24]. In breast cancer cells, elevated levels of BST-2 have been associated with increased cancer cell migration [24,25,26], invasion [24,26,27], adhesion, and resistance to apoptosis/anoikis [28,29]. In addition, it has been suggested that increased immune cell adhesion and resistance of cancer cells to tamoxifen-induced apoptosis is linked to BST-2 expression [27,28,30]. We have also shown that the silencing of BST-2 in murine and human breast cancer cell lines results in a shift from a highly aggressive to a non-aggressive phenotype, including loss of cell to cell and cell to ECM adhesion [24,29], decrease in anchorage-independent growth/survival, formation of invadopodia for ECM remodeling, migration, and invasion [31]. Together, these findings suggest a potential for BST-2 as a valuable therapeutic target. 

In light of the oncogenic roles of BST-2 and its structural characteristics, we developed the first BST-2-based peptide series―B49/B49Mod1. The B49/B49Mod1 peptide series display potent inhibition of adhesion-dependent biological events in breast cancer cells [32,33]. The anti-adhesion B49/B49Mod1 peptides were designed based on the conserved sequence of the ECD of BST-2 [32]. In our previous study, the peptides were shown to block the interaction between breast cancer cells themselves, and between cancer cells and the ECM protein—fibronectin. In addition, the peptide series inhibit cancer cell spheroid-formation, block the interaction of monocytes with cancer cells, and inhibit anchorage-independent growth, in addition to inhibiting tumor growth in the 4T1 mouse model of breast cancer [32]. However, the application of the B49/B49Mod1 peptide series is limited by its size. 

The aim of the present study was to use sequence/structure modification and bioactivity guided separation of B49Mod1 to develop a shorter B49Mod1 analog. Based on the structural information of B49Mod1, which contains three cysteine residues (9CYS, 19CYS, 47CYS, corresponding to 53CYS, 63CYS, and 91CYS in BST-2) and the importance of the BST-2 ECD cysteine residues, especially 91CYS, on cancer cell adhesion [29], we used trypsin-mediated degradation studies to truncate B49Mod1 into six smaller fragments as the first step of the structure-activity relationship (SAR) studies. Three of the predicted B49Mod1 peptide fragments that contain cysteine residues were synthesized. The effect of the peptides on cancer cell adhesion was analyzed. Based on their anti-adhesion potency, the longest fragment of 18 amino acids hereafter called B18 was selected as candidate peptide for further research and development. The identification of a shorter bioactive fragment of the B49/B49Mod1 peptide series for the interruption of cancer cell adhesion may provide insight into the regulation of BST-2 and its interaction with cancer cells.

## 2. Results

### 2.1. Structural Information of B49Mod1

**Figure 1:**
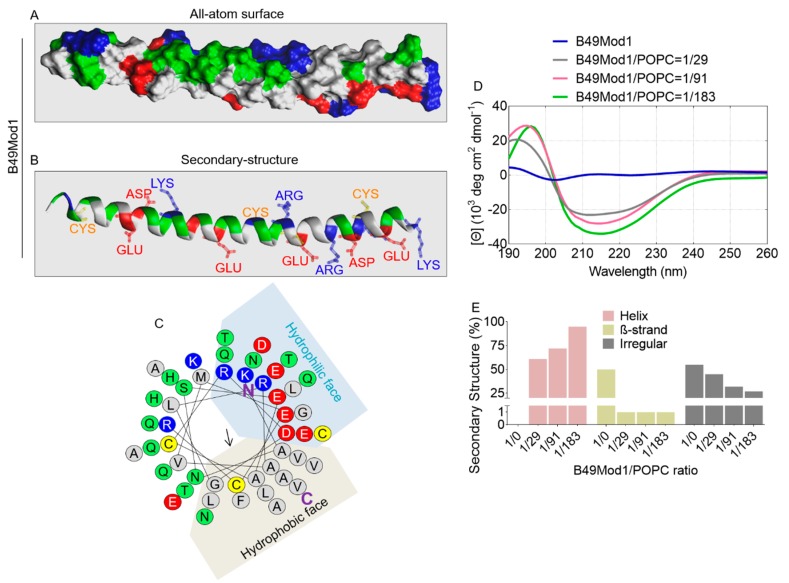
B49Mod1 structural information. (**A**) All-atom surface and (**B**) Secondary structure with thirteen residues’ side chains (GLU, ASP, ARG, LYS, and CYS). (**C**) Helical wheel projection of B49Mod1 core sequence, **N**: N-terminus and **C**: C-terminus. Color code for A-C: red: acidic residue, blue: basic residue, green: polar residue, white: non-polar residue and yellow: CYS residue. (**D**) Circular dichroism (CD) spectrum of B49Mod1 in water or in different concentrations of POPC lipid solution. (**E**) Predicted secondary structures based on the CD analysis.

The sequence of B49Mod1 has been previously described [33]. Here, we used PEP-FOLD3 (http://bioserv.rpbs.univ-paris-diderot.fr/services/PEP-FOLD3/), a web-server aimed at predicting peptide structures from amino acid sequences [34], to predict the structure of B49Mod1 (Figure 1A,B). The all-atom surface structure reveals a spiral or rodlike structure (Figure 1A), supported by the α helix secondary structure (Figure 1B), which reveals the presence of four positive charged residues (blue), six negative charged residues (red), and three cysteine residues (yellow) (Figure 1B). The hydrophobicity (H) and hydrophobic moment (μH) of B49Mod1 are 0.206 kcal/mol and 0.244 kcal/mol, respectively, with α-helicity of 96%. Noteworthy is that B49Mod1 forms an amphiphilic helix with 13 hydrophobic residues on the hydrophobic face and 14 polar/charged residues on the other, a characteristic observed in membrane interacting peptides [35,36]. This observation was further shown by helical wheel projection (Figure 1C) using HeliQuest (http://heliquest.ipmc.cnrs.fr/) [37,38,39]. Circular dichroism (CD) spectrum shows that the peptide adopts different secondary structures in different conditions, as predicted by the CD spectrum (https://capito.uni-jena.de/index.php). In water, the peptide adopts a random/flexible structure with 50% β-strand (Figure 1D,E). When mixed with 1-Palmitoyl-2-oleoyl-sn-glycero-3-phosphocholine (POPC) lipid solution, the CD spectrum showed a positive amplitude around 200 nm and a shift of the negative amplitude at the region of 210–230 nm (Figure 1D), indicating the formation of helical structure [40]. Indeed, the predicted helicity increased to 61%, 72%, and 95% with peptide/lipid ratios of 1/29, 1/91, and 1/183, respectively (Figure 1E). In addition, the β-strand structure decreased to 1% when the peptide stays in the lipid environment. These data reveal the structure of B49Mod1 and highlight the importance of the lipid membrane in the modulation of the B49Mod1 structure. 

### 2.2. Cysteine-Linked Disulfide Bond is Important for Anti-Adhesion Activity of B49Mod1 

**Figure 2:**
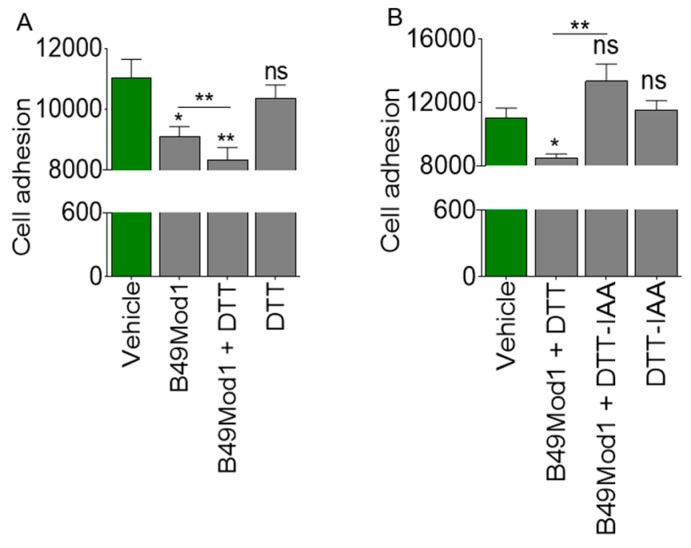
Disulfide (S-S) bond plays a role in B49Mod1-mediated inhibition of cell adhesion. (**A**) Adhesion of 4T1 cells was inhibited by B49Mod1 in the presence of unquenched S-S reducing agent 1,4-Dithiothreitol (DTT). (**B**) Adhesion of 4T1 cells was rescued in reactions in which free cysteine residues were alkylated by the Iodoacetic acid (IAA) following the reduction of S-S by DTT. Experiments were repeated three times with similar results. Error bars represent S.E.M. Ordinary one-way ANOVA test (Dunnett’s correction), and two-tailed t-test (Welch’s correction) were used to determine the differences between the groups. *p < 0.05, **p < 0.01, and ns = non-significant.

It has been previously shown that B49 and B49Mod1 possess anti-adhesion activity [32]. Since B49Mod1 contains cysteine residues that mediate BST-2 dimerization [12,13,14] and the adhesion-promoting function of BST-2 [29], we examined the role of cysteine-linked disulfide (S–S) bonds in the anti-adhesion function of B49Mod1. 2 mol equivalents of 1,4-Dithiothreitol (DTT), a reducing agent that reversibly breaks down protein S–S was added to B49Mod1 without quenching the oxidative process followed by incubation at 56° C for 30 minutes. In a different reaction, Iodoacetic acid (IAA), an alkylating agent that reacts with cysteine residues, was added to the B49Mod1-DTT reaction to quench oxidation of S–S bonds. These reactions were used in cell adhesion assays. In these assays, B49Mod1 with the reducing agent―DTT and without the alkylating IAA retained the ability to inhibit cell adhesion and exhibited a significant change from peptide alone (Figure 2A). However, reducing S–S linkages with DTT and alkylating (with IAA) free cysteine in B49Mod1 abrogates its anti-adhesion ability and rescues adhesion (Figure 2B). This result indicates that eliminating S–S bonds may destroy the natural conformation of B49Mod1, resulting in loss of activity. By reducing S–S in B49Mod1 and arresting the reaction, it was possible to characterize the effect of loss of S–S on peptide function. However, it is unknown whether the original S-S bond and additional S-S intermediates were formed in unquenched reactions to result in the inhibition observed in Figure 2A (3^rd^ bar). Moreover, the relationship between the B49Mod1 conformation and S–S bond formation was not established in this reaction. 

### 2.3. Extended Incubation Time Results in Loss of B49Mod1 Activity 

**Figure 3:**
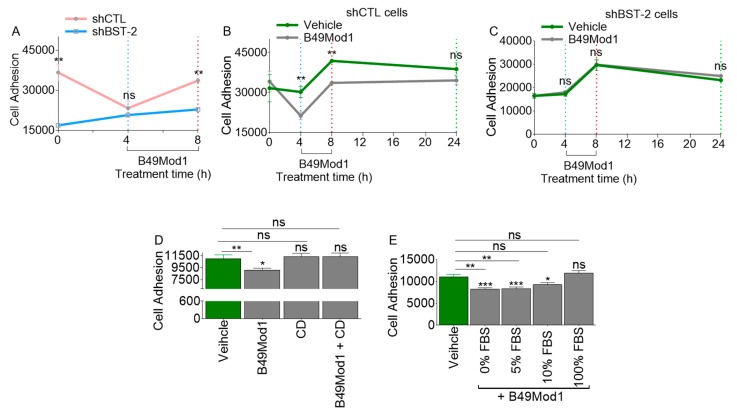
B49Mod1 loses potency upon extended incubation times. (**A**) Adhesion of PKH67 Green-labeled shCTL and shBST-2 4T1 cells onto B49Mod1-treated 4T1 shCTL monolayers at 0, 4, and 8 hours. (**B**) Adhesion of PKH67 Green-labeled shCTL 4T1 cells onto Vehicle or B49Mod1-treated shCTL monolayers at 0, 4, 8 and 24h. (**C**) Adhesion of PKH67 Green-labeled shBST-2 4T1 cells onto Vehicle or B49Mod1 treated shBST-2 monolayers at 0, 4, 8, and 24 h. (**D**) Adhesion of PKH67 Green-labeled shCTL 4T1 cells onto shCTL 4T1 cells pre-treated with MDA-MB-231 conditioned media (CM) overnight at 37 °C. (**E**) Adhesion of PKH67 Green-labeled shCTL 4T1 cells onto shCTL 4T1 monolayer treated with B49Mod1 that was pre-incubated with Roswell Park Memorial Institute (RPMI) 1640 media containing different concentrations of serum (0%, 5%, 10%, 100%) overnight at 37° C. Experiments were repeated three times with similar results. Error bars represent S.E.M. Ordinary one-way ANOVA test (Dunnett’s correction), and two-tailed t-test (Welch’s correction) were used to determine the differences between the groups. *p < 0.05, **p < 0.01, ***p < 0.001, and ns = non-significant.

Here we examined the kinetics of B49Mod1-mediated inhibition of adhesion. First, monolayers of BST-2-expressing 4T1-shCTL cells were treated with vehicle (0 h) or B49Mod1 for 4 h and 8 h. The excess peptide was washed off, and equivalent numbers of PKH67Green-labelled 4T1-shCTL and shBST-2 (BST-2-suppressed) cells were added to the B49Mod1-treated 4T1-shCTL monolayers. Analysis of adhesion [24,29] showed that suppression of BST-2 inhibits adhesion as expected (Figure 3A, time point 0). Strikingly, B49Mod1 at 4 h blocked adhesion to a similar level as suppression of BST-2 (Figure 3A, blue vertical dotted lines). In contrast, while adhesion remained inhibited in shBST-2 cells at the 8 h time point, we observed a rebound of adhesion to untreated level in shCTL cells treated with B49Mod1 at 8 h (Figure 3A, red vertical dotted lines), indicating loss of peptide potency. Next, we compared adhesion of control, and B49Mod1-treated shCTL cells over time following treatment of shCTL monolayers with B49Mod1 for 4 h and 8 h. Control cells were treated with vehicle (0 h). The highest B49Mod1-mediated inhibition of adhesion occurred at 4 h (Figure 3B, blue vertical dotted lines). After which a rebound of adhesion was observed by the 8 h time point, although not to the level of control peptide-treated cells (Figure 3B, red vertical dotted lines). Further extension of incubation time to 24 h results in complete abrogation of the anti-adhesion effect of B49Mod1 (Figure 3B, green dotted lines). The rebound in shCTL cell adhesion at 8 and 24 h time points may be a combination of loss of peptide potency over long incubation times and rapid proliferation rate of 4T1 cells. Importantly, the treatment of shBST-2 cells with B49Mod1 did not change the pattern of cell adhesion, indicating that B49Mod1 may mostly inhibit BST-2-mediated cancer cell adhesion (Figure 3C). Additionally, the data reveal that B49Mod1 may be susceptible to proteolytic deactivation, and therefore, a need for stable B49Mod1 analogs. 

### 2.4. B49Mod1 is Susceptible to Proteolysis

Data presented in Figure 3A,B suggest that B49Mod1 may be susceptible to proteolysis, which may be the cause of loss of activity (potency) on extended incubation times on cells. This prediction was tested by analyzing the stability of B49Mod1 in conditioned culture media from cancer cells, or in fetal bovine serum (FBS) to simulate the proteolytic activity that may be present in vivo and in the tumor microenvironment. Briefly, 200 ng/well B49Mod1 was incubated overnight at 37 °C with either MDA-MB-231 conditioned media (CM) or with Roswell Park Memorial Institute (RPMI) 1640 media containing different concentrations of serum (0%, 5%, 10%, 100%). CM-treated B49Mod1 or serum-treated B49Mod1 were added to shCTL 4T1 cell monolayers and incubated at 37 °C for 4 h. The excess peptide was removed, and PKH67 Green-labeled shCTL 4T1 cells were added to the B49Mod1-treated monolayers, and adhesion was assessed after 4 h. CM without B49Mod1 was used as control. Treatment of B49Mod1 with CM deactivates B49Mod1 and results in the loss of anti-adhesion activity (Figure 3D). Similarly, incubation of B49Mod1 in the FBS alone deactivates the peptide and results in loss of activity (Figure 3E).

### 2.5. Trypsin Proteolysis of B49Mod1

**Figure 4:**
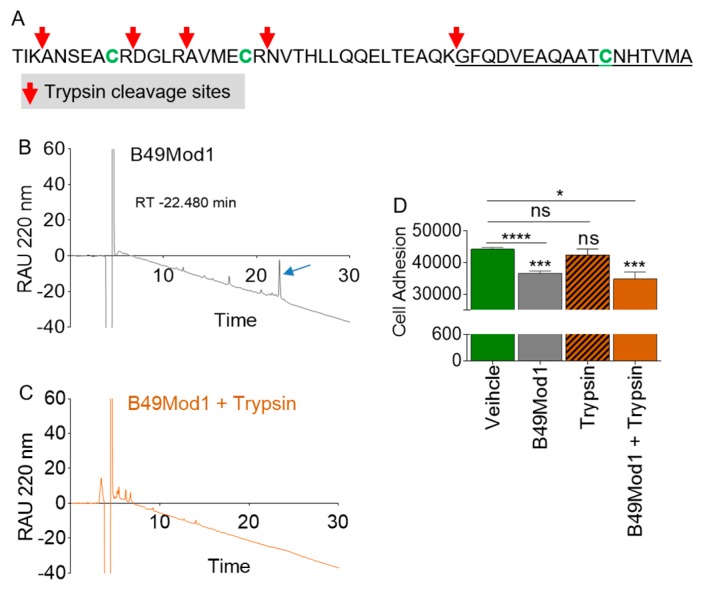
Proteolytic digestion of B49Mod1 using trypsin. (**A**) B49Mod1 sequence and predicted trypsin cleavage sites (red arrows). Reversed-phase high-performance liquid chromatography (RP-HPLC) analysis of (**B**) undigested B49Mod1 and (**C**) trypsin-digested B49Mod1. (**D**) Adhesion of 4T1 cells atop 4T1 monolayers treated with B49Mod1 or trypsin-digested B49Mod1. Experiments were repeated three times with similar results. Error bars represent S.E.M. Ordinary one-way ANOVA test (Dunnett’s correction), and two-tailed t-test (Welch’s correction) were used to determine the differences between the groups. *p < 0.05, ***p < 0.001, ****p < 0.0001, and ns, non-significant.

Here, we aim to determine how the serine protease - trypsin degrades B49Mod1. A schematic prediction of trypsin cleavage sites of B49Mod1 (Figure 4A, red arrows) is shown with three of tryptic fragments containing cysteine residues (Figure 4A). Since trypsin can cut B49Mod1 into six fragments (Figure 4A), the peptide was treated with trypsin, as detailed in the methods section. Following treatment, trypsin digests were analyzed by reverse-phase high-performance liquid chromatography (RP-HPLC). Trypsin treatment of B49Mod1 (Figure 4B, blue arrow) results in complete degradation of B49Mod1 as evidenced by the absence of a B49Mod1 peak in the chromatogram (Figure 4C). Interestingly, the trypsinolysis of B49Mod1 does not have an effect on peptide activity (Figure 4D). The reason for the retained anti-adhesion activity of trypsin-digested B49Mod1 is unknown but may be related to the presence of one or all of the three B49Mod1 cysteine residues in three different fragments. One of the fragments B18 contains 12CYS, which in B49Mod1 is 47CYS, and in BST-2 is 91CYC. In BST-2, 91CYS is indispensable for BST-2 -mediated cell adhesion [29]. The sequence information and characteristics of predicted tryptic fragments are shown in Table 1.

### 2.6. Identification of the Smallest and Most Potent B49Mod1 Fragment

**Figure 5:**
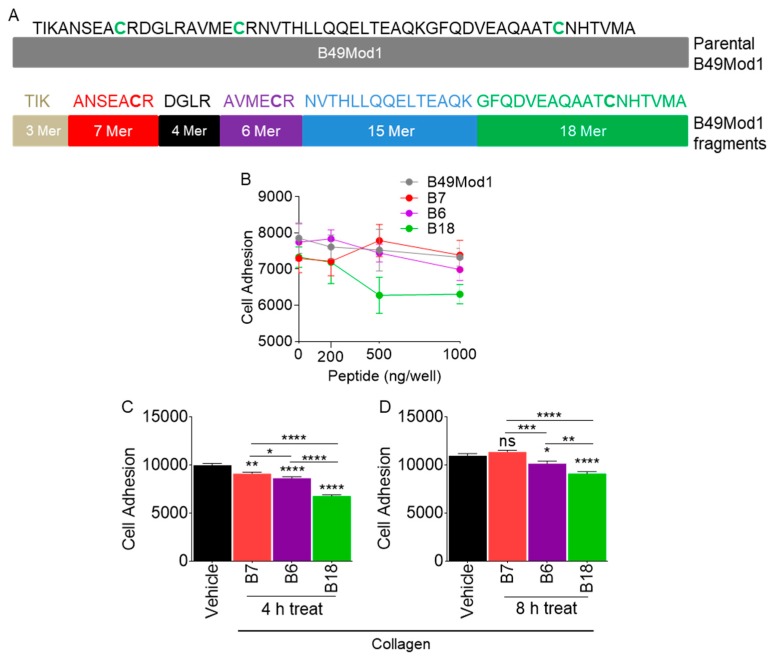
Identification of the smallest and most potent B49Mod1 fragment. (**A**) Schematic of B49Mod1 and its fragments. (**B**) Adhesion of shCTL 4T1 cells pre-treated with different concentrations of B7, B6, and B18 peptides on fibronectin-coated wells. (**C**,**D**) Adhesion of shCTL 4T1 cells pre-treated with different concentrations of B7, B6, and B18 peptides on collagen-coated wells. Experiments were repeated three times with similar results. Error bars represented S.E.M. Ordinary one-way ANOVA test (Dunnett’s correction), and two-tailed t-test (Welch’s correction) were used to determine differences between groups. *p < 0.05, **p < 0.01, ***p < 0.001, ****p < 0.0001, and ns = non-significant.

Trypsin is a highly specific serine protease that cleaves at the carboxyl side of lysine and arginine residues. As shown in Figure 4B,C, trypsin completely degrades B49Mod1 while retaining anti-adhesion activity (Figure 4D). As a result, we used trypsin as a tool to identify the smallest and most potent B49Mod1 fragments. The proteolysis analyses illustrated in Figure 4B,C provides the basis for identifying a shorter B49Mod1 fragment, that retains the anti-adhesion activity of the full-length B49Mod1. Indeed, the analysis of the B49Mod1 sequence shows that trypsin can cleave the peptide into six overlapping fragments of 3, 7, 4, 6, 15, and the longest of which is the C-terminal peptide sequence of 18 amino acids (Table 1 and Figure 5A). We synthesized the three peptides containing the B49Mod1 cysteine residues, including the 7 Mer (B7), 6 Mer (B6), and 18 Mer (B18). Cancer cell adhesion assay identified B18 as superior to B49Mod1 (Figure 5B). Additionally, inhibition of cancer cell adhesion by B18 was more significant compared to B7 and B6, and all peptides, especially B18 was more effective at 4 h post-treatment (Figure 5C) compared to 8 h post-treatment (Figure 5D). These data suggest that B18 is the shortest analog that retains the anti-adhesion profile of B49Mod1.

### 2.7. All-Atom and Secondary Structure of B18

**Figure 6:**
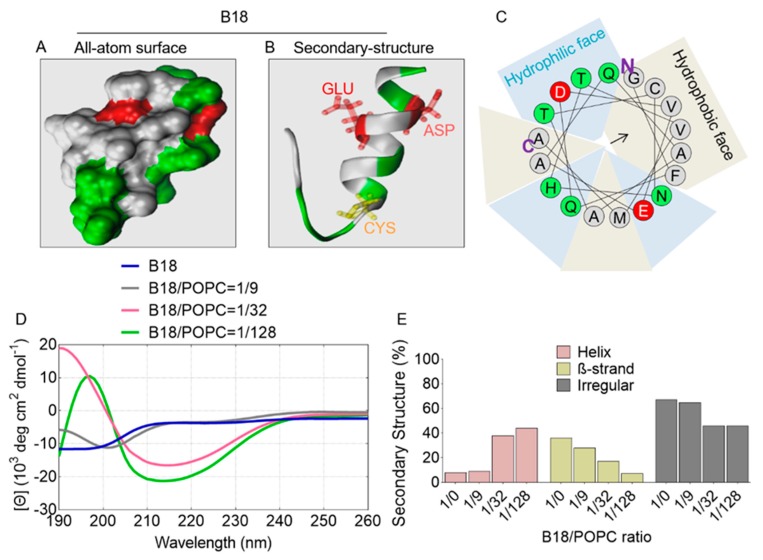
Predicted structure of B18 peptide. (**A**) All-atom surface. (**B**) Secondary structure with three residues’ side chains (GLU, ASP, CYS). (**C**) Helical wheel projection of B18, **N**: N-terminus, and **C**: C-terminus. Color code: red: acidic residue, green: polar residue, white: non-polar residue and yellow: CYS residue. (**D**) Circular dichroism (CD) of B18 in water or in different concentrations of POPC lipid solution. (**E**) Predicted secondary structure based on the CD analysis.

For insight into the biophysical chemistry of B18, we used PEP-FOLD3 to predict peptide secondary structure, as shown in Figure 6. B18 bears two acidic residues (GLU and ASP), resulting in a net negative charge of -2. The H and μH are 0.358 kcal/mol and 0.222 kcal/mol, respectively, with α-helicity of 56%. In addition, B18 adopts a V-shaped structure with a kink around the 13^th^ residue ASN, and an end-to-end distance of ~1.5 nm. With relatively lower μH than B49Mod1, B18 shows a mixed distribution of hydrophobic and hydrophilic residues as depicted by the helical wheel of the full sequence (Figure 6C). CD analysis reveals that similar to B49Mod1, B18 is a random coil in water, with minor β-strand character (Figure 6D,E). However, the peptide adopts a helix/random coil conformational state in different lipid concentrations with increasing helix and decreasing random coil features as the lipid concentration increases. As shown in Figure 6D,E, B18 helicity increased from 9% to 37%, and to 44% when the peptide/lipid ratio changed from 1/9 to 1/32, and 1/128, respectively (Figure 6E). The increase in peptide helicity is associated with a decrease in β-strand and random coil features. Further validation for the B18 structure was provided by APD3: Antimicrobial Peptide Calculator and Predictor (http://aps.unmc.edu/AP/) [41] a prediction website. APD3 predicts that B18 may form alpha helices and that the peptide may interact with the membranes. These results reveal the dependence of the B18 structure on lipid concentration. 

### 2.8. B18 Binds BST-2

**Figure 7:**
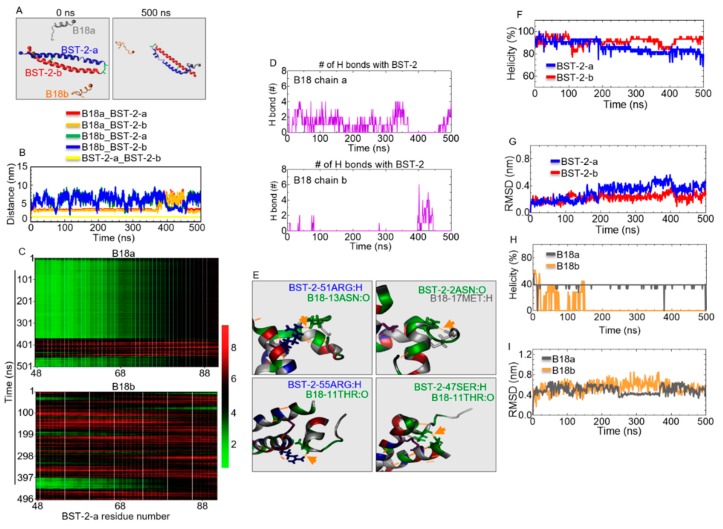
Predicted potential interaction between B18 peptides with the extracellular domain of BST-2. (**A**) Initial (t = 0 ns) and final (t = 500 ns) snapshots of MD simulation. Color code: blue: BST-2 chain a, red: BST-2 chain b, gray: B18 chain a, orange: B18 chain b. (**B**) Evolution of center of mass (COM) distance between each chain in the system. (**C**) COM distance of B18 chain a (top) and B18 chain b (bottom) with COM of BST-2 chain a protein as a function of time. Green means binding, and red means non-binding. (**D**) The number of hydrogen bonds (H bonds) of B18 chain a (top) and B18 chain b (bottom) with BST-2 protein as a function of time. (**E**) Representative images of hydrogen bonds (orange arrows) between B18 peptides and BST-2 protein. (**F**) The helicity of BST-2 chains as a function of time. (**G**) Root-mean-square deviation (RMSD) of BST-2 chains as a function of time. (**H**) The helicity of B18 chains as a function of time. (**I**) RMSD of B18 chains as a function of time.

Since B18 is an analog of B49Mod1, which is derived from the ECD of BST-2, we sought to determine if B18 interacts with BST-2. Thus, we assessed the structural organization of B18 on BST-2 using sequences (residues 48 to 91) from the ECD of BST-2. MD simulations of two chains of B18 (chains a and b) and BST-2 (BST-2 chains a and b, Figure 7A) in 0.15 M KCl aqueous solution were performed for 500 ns duration. The B18 peptides were initially placed in the same distance (5 nm) to the center of BST-2 protein (Figure 7A, left). The 500 ns snapshot depicts the binding of B18 chains a and b onto BST-2 protein (Figure 7A, right). The distance between molecules is a direct way to assess whether the interaction is occurring [42]. The evolution of distances between the center of mass (COM) of each B18 peptide to the COM of each BST-2 chain shows that binding occurs mainly with one of the B18 chains, while the other chain moves freely in the aqueous solution as reflected by the degree of fluctuations (Figure 7B). Binding times were 5–360 ns and 490–500 ns for B18 chain a onto BST-2 (Figure 7B, red and orange lines) and 400–450 ns for B18 chain b onto BST-2 (Figure 7B, green and blue lines). During these B18•BST-2 bindings, the BST-2 chains were tightly associated with each other (Figure 7B, yellow lines).

### 2.9. Identification of B18 Binding Sites

To further explore the structural and dynamic basis for the binding of B18 to BST-2, we calculated the distance between the COM of B18 chain a and chain b and the COM of individual residues of BST-2-a as a function of time as represented by the heat maps (Figure 7C, green denotes binding, red shows unbinding). Binding affinity between B18 chains a and b for BST-2 were higher near the N-terminus of BST-2—around residues 48 to 67 (Figure 7C, green region). The number of hydrogen bonds between each B18 chain to the BST-2 as a function of time was calculated (Figure 7D). The hydrogen bond was determined based on the cutoffs for the angle Hydrogen-Donor–Acceptor (30°) and the distance between Donor and Acceptor (0.35 nm). The existence of hydrogen bonds further implies the occurrence of the binding event. The number of hydrogen bonds between individual residues of B18-a with BST-2 over the simulation time was determined (Figure S1). Residues such as 1GLY, 7ALA, 10ALA, 11THR, 12 CYS, 14HSD show more hydrogen bonds than other residues (Figure S1). Representative snapshots of B18•BST-2 binding interactions show the presence of hydrogen bonds between BST-2-55ARG: atom hydrogen (H) and B18-11THR: atom oxygen (O); BST-2-47SER:H and B18-11THR:O; BST-2-51ARG:H and B18-13ASN:O; BST-2-2ASN:O and B18-17MET:H (Figure 7E).

### 2.10. Structural Properties of B18 and BST-2 upon Binding

Simulations were based on two full helical structures of BST-2 (Figure 7F). Both chains a and b of BST-2 maintained their helical structure over 70% from 0 to 460 ns. However, chain a unfolded part of the helical structure from 460 ns, and the helicity dropped to 65% at 500 ns (Figure 7F, blue line), which could be due to its interaction with B18 peptides (Figure 7A, right). RMSD of BST-2 chains (Figure 7G) shows that BST-2 chain b is more stable than BST-2 chain a as indicated by an RMSD of ~0.2 nm for chain b compared to ~0.4 nm for chain a, which is consistent with its helicity trend (Figure 7F). Secondary structure analysis indicates that B18 chain a is ~40% helical, while chain b lost all helical structure, perhaps due to less binding (Figure 7H). RMSD analysis shows that while B18 chains a and b fluctuate over 0.4 nm, chain a was stable for a short time at ~250 to 360 ns (Figure 7I, gray line). These results provide atomic-level insight into B18•BST-2 interaction.

### 2.11. B18 Binds Zwitterionic Membrane

**Figure 8:**
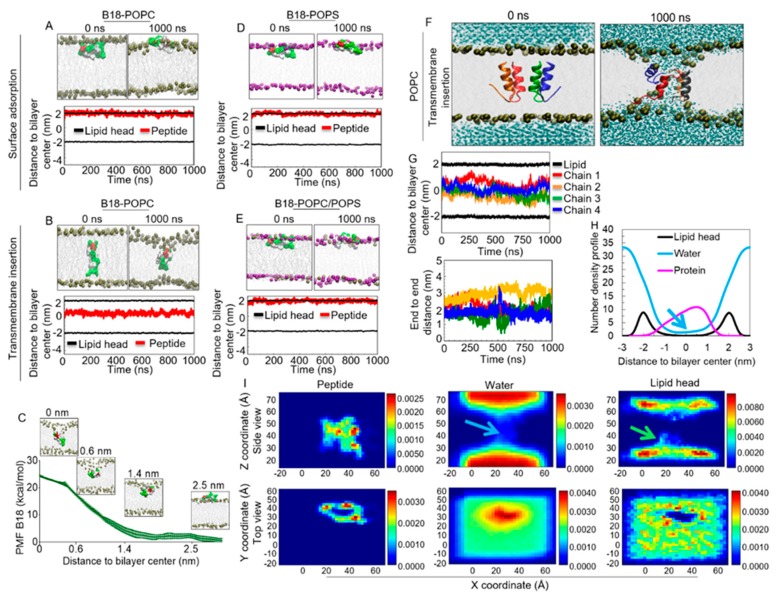
MD simulations show potential interaction between B18 with model cell membranes. (**A**) Top: initial (t = 0 ns) and final (t = 1000 ns) snapshots of a single B18 peptide atop 1-palmitoyl-2-oleoyl-sn-glycero-3-phosphocholine (POPC) lipid bilayer. Bottom: COM distance between B18 peptide and lipid bilayer as a function of time. (**B**) Top: initial (t = 0 ns) and final (t = 1000 ns) snapshots of a single B18 peptide in the center of POPC lipid bilayer. Bottom: COM distance between B18 peptide and lipid bilayer as a function of time. (**C**) Potential of mean force of transferring B18 peptide from the membrane surface (x = 2.5 nm) to the membrane center (x = 0 nm). (**D**) Top: initial (t = 0 ns) and final (t = 1000 ns) snapshots of a single B18 peptide atop 1-palmitoyl-2-oleoyl-sn-glycero-3-phospho-L-serine (POPS) lipid bilayer. Bottom: COM distance between B18 peptide and lipid bilayer as a function of time. (**E**) Top: initial (t = 0 ns) and final (t = 1000 ns) snapshots of a single B18 peptide atop POPC/POPS mixed lipid bilayer (POPC:POPS = 3:1). Bottom: COM distance between B18 peptide and lipid bilayer as a function of time. (**F**) Initial (t = 0 ns) and final (t = 1000 ns) snapshots of four B18 peptide chains inside of POPC lipid bilayer (**G**) Top: COM distance between each B18 chain and POPC lipid bilayer. Bottom: End to end distance of each B18 chain as a function of time. (**H**) Density profile of Lipid bilayer, water, and B18 peptides. (**I**) Two-dimensional (2D) density profile of peptide, water, and lipid head from side view (top row) and top view (bottom row). Blue arrows indicate water channel formation, while green arrow points to the deformed membrane.

Aside from the interaction with BST-2, membrane binding is critical for specific targeting and functions of peptides with anti-cancer activities. Thus, we sought to understand how B18 interacts with the cell membrane. Two independent MD simulations were carried out for B18 to determine the mechanism of peptide interaction with a model zwitterionic POPC lipid bilayer. The simulations were performed using systems descriptions and setups that were described in the methods sections modified from previously described protocols [43,44,45,46,47,48,49,50,51,52,53,54]. Briefly, single peptides were initially placed either on the surface or in the transmembrane of the POPC lipid bilayer. The system was first minimized and equilibrated, followed by a 1000 ns production MD simulation. The single peptide simulations depict the ability of B18 to interact with the membranes when surface-adsorbed versus when transmembrane-inserted. For surface simulation, B18 stably binds the membrane surface over the 1000 ns (Figure 8A). In the transmembrane simulation, B18 remained in the center of the membrane for the duration (1000 ns) of the simulation (Figure 8B).

### 2.12. Energetics of B18 Insertion into the POPC Lipid Bilayers

To assess the energetics of the transfer of the peptides from the aqueous phase into lipid bilayers, we used the umbrella sampling MD simulation to determine the potential of mean force (PMF) or free energy profile for the peptide transfer from the membrane surface to the membrane center. Three independent determinations of the free energy profiles as a function of the distance between the peptide and the bilayer center were performed. Convergence of the PMF calculation was attested by the free energy values calculated in different windows of the simulations (Figure 8C). Results show a sizable free energy barrier of approximately 24.29 ± 0.33 kcal/mol (the y value at x = 0) for peptide insertion into the lipid bilayer. It was observed that when B18 was close to the center of the membrane (~0.39 nm), the energy profile exhibits a shoulder with 21.51 ± 0.52 kcal/mol (Figure 8C, red arrow). This observation implies that the center of lipid bilayer may be the second favorable position for B18.

### 2.13. B18 Binds Anionic POPS Lipid Bilayer

The membrane of cancer cells is known to be more negatively charged than normal cells [55]. As a result, the simulation of a single peptide with a POPS or POPC/POPS mixed bilayer (lipid ratio = 3:1) were performed to investigate the interaction between B18 and other membrane types with the net negative charge. Similar to the observation made with POPC, B18 stably binds both POPS (Figure 8D) and POPC/POPS mixed lipid bilayer (Figure 8E) throughout the simulation time. Together, these data suggest that B18 has the property of membrane interacting peptides. 

### 2.14. B18 forms Transmembrane Pores

Simulations of four monomer chains of B18 on the POPC lipid bilayer show that the peptides were initially (t = 0 ns) arranged with the hydrophilic residues facing each other while most hydrophobic residues face the lipid tails (Figure 8F, left). Within 1000 ns simulation, intermolecular interactions occur, and the four peptides were surrounded by a cavity as an aggregate. The cavity was continuously filled with water molecules, indicating potential water channel or pore formation (Figure 8F). Furthermore, the phosphate lipid heads bend into the aggregate to stabilize the water channel, indicating that the pore structure is toroidal (membrane pore formed by peptides and lipid heads). A detailed analysis of the evolutions of the distances between COM of each B18 chain and lipid bilayer indicates that the peptides stayed inside the lipid bilayer within the simulation time and that the COM of the peptides were at the region of -1 to 1 nm, which is the central region of the lipid bilayer (Figure 8G, top). We also analyzed end to end distance of each peptide (Figure 8G, bottom). Initially, all the peptides bear a “V-shaped” kink with the end to end distance at ~1.5 nm (Figure 8G, Figure S2A). As the simulation progressed, chains 1, 3, and 4 retained the “V-shape” with end to end distance of ~1.8 nm, while chain 2 unfolded to ~3 nm (Figure 8G, Figure S2A). The RMSD evolution of each peptide shows that the structures of chain 1 and chain 2 were less stable than the other two peptides (Figure S2B), as their average RMSD were above 0.4 nm. Further analysis reveals that peptide helicity was maintained between 40% to 60% (Figure S2C). The formation of hydrogen bonds between the peptide chains provides additional evidence that the peptides were associated as a bundle inside the membrane (Figure S3A). Representative snapshots of B18•membrane binding interactions show the presence of hydrogen bonds between Chain1-8GLN:H and Chain2-6GLU:O, Chain2-13ASN:O and Chain3-15THR:H, Chain3-8GLN:H and Chain4-16VAL:O, Chain4-1GLY:H and Chain1-18ALA:O (Figure S3B). It is noted that B18 peptide chains also formed hydrogen bonds with both the membrane and water (Figure S3C), although the average number of hydrogen bonds were not significantly different between each peptide chain with membrane or with water. These peptide behaviors suggest that the peptide chains were adjusting their structure to maintain the pore inside the membrane. 

In addition, the last 50 ns density profile of total peptides (Figure 8H, magenta line; Figure 8I, top left) also validates the position of the peptides. Water channel formation has been used as an indicator of pore formation for years [56,57,58,59]. In our MD system, both one-dimensional (1D) density profiles of water and lipid heads (Figure 8H, blue and black lines) and two-dimensional (2D) density plots (Figure 8I) clearly show the formation of water channels and membrane deformation. This is in contrast to normal lipid bilayer that will not have water in the center of lipid bilayer [60]. Therefore, this indicates that B18 peptides have the potential to impair the cell membrane and form pores in the cell membrane.

Lipid heads are usually distributed on the membrane surface due to their hydrophilic properties that are reflected by two symmetric distributions of −2.5–−1.2 nm and 1.2–2.5 nm [61]. In our analysis, membrane deformation results in the bending of lipid heads inwards towards the center of the lipid bilayer (Figure 8H,I, top right: green arrow). The top view of the 2D density profile further confirms that the pore was surrounded by the peptides and filled with water molecules (Figure 8I, bottom). Together, these MD data highlight the potential that B18 may assemble and form pores that may impair membrane integrity, ultimately inhibiting cancer cell adhesion. 

### 2.15. Polyfunctional Effects of B18 on Cell Viability

**Figure 9:**
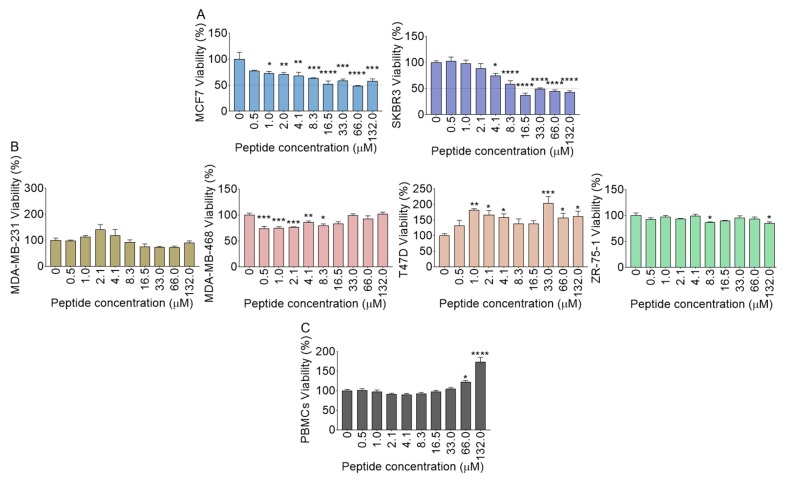
Effect of B18 on cell viability by MTT assay. (**A**) MCF7 and SKBR3 viability. (**B**) MDA-MB-231, MDA-MB-468, T47D, ZR-75-1, and (**C**) PBMCs viability. Experiments were repeated three times with similar results. Error bars represent S.E.M. Ordinary one-way ANOVA test (Dunnett’s correction) was used to determine the differences between each peptide concentration and vehicle (Peptide concentration = 0), whereas *p < 0.05, ** *p* < 0.01, ***p < 0.001, ****p < 0.0001 and non-significant was not shown for clarity.

The cytotoxic effect of B18 against various human breast cancer cell lines was evaluated at different concentrations by MTT assay. Following 24 h incubation of cells with B18, MCF7 and SKBR3 cell lines showed significant reductions in cell viability with high half-maximal inhibitory concentration (IC_50_) of 59.8 ± 5.3 µM for MCF7 and moderate IC_50_ of 11.2 ± 3.4 µM for SKBR3 (Figure 9A). In contrast to MCF7 and SKBR3 cells, we were unable to calculate B18 IC_50_ values for MDA-MB 231, MDA-MB 468, T47D, and ZR-75-1 cell lines as we did not observe any significant loss of viability, even at the highest peptide concentration (132.0 µM) tested (Figure 9B). When quiescent human peripheral blood mononuclear cells (PBMCs) were treated with B18, no cytotoxic effect was observed (Figure 9C). However, at concentrations of 66 and 132 µM, B18 enhanced the viability of PBMCs in a concentration-dependent manner (Figure 9C). Although the reason for increased PBMCs viability in the presence of B18 was not explored, it is beyond dispute that the peptide effects may largely vary among different cell types. For example, subtle increases in viability were observed in T47D at different B18 concentrations. Thus, the increased T47D or PBMCs viability may suggest that B18 may affect the behavior of these cells in various ways.

## 3. Discussion

We have previously shown that B49Mod1 is a BST-2-based peptide that inhibits cancer cell adhesion [32]. In this study, we show that a cysteine-linked disulfide bond is important for the anti-adhesion activity of B49Mod1. Although the mechanism of B49Mod1-mediated blocked of cell adhesion is not known and is not the focus of this study, it is known that the BST-2 ECD contains three cysteine residues that are involved in BST-2 dimerization [12,13,14]. The last cysteine residue at position 91 is indispensable for BST-2-mediated promotion of cancer cell adhesion [29], and B49Mod1 (which contains three cysteine residues) may target the cysteine residues in the ECD of BST-2. Additionally, although B49Mod1 is effective in blocking cancer cell adhesion, we found that the peptide is susceptible to deactivation by serum and cellular proteases, which in turn may reduce its potency. Since B49Mod1 is a long peptide and the activity of lengthy peptides is known to be hindered by the loss of bioactive structure and rapid proteolysis [62], we embarked on developing a short B49Mod1 analog.

Here we used bioactivity-guided separation to identify B18 as the minimal peptide sequence mediating the anti-adhesion activity of B49Mod1. CD spectra of B18 and the parental B49Mod1 show that structural alteration upon reaction with POPC lipid is a common feature of the peptides. Indeed, both peptides underwent significant alterations to their secondary structure following incubation with varying the concentration of POPC. In general, however, helical features increased in both peptides with increasing lipid concentration. 

In addition, atomistic MD simulations were used to predict the association of B18 with the BST-2 ECD and with the cell membranes. MD simulations allowed determination of the interaction between B18 and BST-2. We observed that B18•BST-2 interaction was stable, with multiple binding and unbinding events observed between two B18 chains and BST-2. A comparison of the simulations describing the binding of two chains of B18 to BST-2 reveals the presence of intermolecular hydrogen bonds. Some of the observed hydrogen bonds include but not limited to BST-2-55ARG:H and B18-11THR:O; BST-2-47SER:H and B18-11THR:O; BST-2-51ARG:H and B18-13ASN:O; BST-2-2ASN:O and B18-17MET:H. These hydrogen bonds may encourage hydrophobic interactions between B18 and BST-2. Analysis of the dynamics of B18 binding to BST-2 reveals that chains a and b of B18 associate with BST-2 at a region near the N-terminus of BST-2. 

Since the N-terminus of the BST-2 ECD is close to the BST-2 transmembrane domain, we explored the mechanism of membrane binding by B18. Peptide interaction with the cell membrane can occur in two different states— surface and transmembrane states [63,64]. Thus, B18 simulations were analyzed in terms of its interactions with the model lipid bilayer and the orientation of the peptide relative to the bilayer. Monomeric B18 was stable on the surface of both zwitterionic and anionic membranes comparably. Detailed MD structural analysis of B18 simulations shows that the peptide structure is altered during binding to the model membrane. In particular, B18 shows distinct changes when monomeric peptides were surface-adsorbed versus when a bundle of four peptides was membrane-inserted into the POPC bilayer. Within the membrane, both single and multiple (four) B18 peptides were stable in the center of the membrane. The bundle of B18 peptides formed pores of the toroidal type, where the peptides and the hydrophilic lipid head groups line the transmembrane water channel with structural assemblies of four peptide chains. The observed pore-forming property of B18 is an indication that B18 may disrupt cell membrane integrity. The B18 pore structure is similar to that of a membrane-active peptide identified from soy protein and known to disrupt membrane integrity [65]. Although B18 bound the lipid bilayer, the binding is modulated by a steep free energy barrier of 24.29 ± 0.33 kcal/mol, suggesting that B18 may not easily insert into the membrane. Indeed, the free energy barrier that B18 needs to overcome to enter the cell membrane is higher than that of melittin, a known membrane lytic peptide [66,67]. The contributors for the change in the total free-energy difference between B18 in the membrane versus B18 in the aqueous phase may include differences in electrostatic and nonpolar effects, lipid perturbation, and peptide immobilization effects, as well as peptide conformational changes, such as transitions from random coil to α-helix and vice versa. 

The energetics of B18 insertion into the lipid bilayers highlights that B18 will benefit from further modification. The structural analysis of B18 was used to identify important features that may be modified in future studies. These include two negatively charged residues (4ASP and 6GLU) and membrane interacting residues such as ALA. The B18 residues such as 4ASP, 6GLU, and 15THR may be substituted to positive residues to enhance interaction with negatively charged cancer cell membranes. ALA may be substituted to LEU to increase peptide H and µH, all of which are important features of membrane interacting peptides. These properties may be leveraged in further optimization of B18. Furthermore, unlike B49Mod1 with three cysteine residues (9CYS, 19CYS, 47CYS) that may contribute to its anti-adhesion property, B18 retains the 47CYS of B49Mod1, which in B18 is 12CYS. This is an important modification because multiple cysteine residues may promote peptide susceptibility to oxidation, which is a major cause of chemical instability. Thus, reducing the numbers of cysteine residues from three in B49Mod1 to one in B18 did not affect the anti-adhesion property of the peptide and may have a beneficial effect. 

As expected, wet-lab experiments show a comparable anti-adhesion function between B18 and B49Mod1. Specifically, compared to B49Mod1, B18 inhibits cell-cell and cell-ECM adhesion, although both peptides lost activity at the 8 h time point (Figure 3B and Figure 5C,D), indicating shorter half-life. These data suggest that B18 is the shortest analog that retains the anti-adhesion property of B49Mod1. In contrast to B49Mod1 [32,33], B18 possesses polyfunctional effects on cells where at high to moderate concentration, the peptide reduced the viability of some cancer cells and had no effect on others. However, the effect on quiescent healthy PBMCs was that of increased cell viability with high peptide concentrations (66.0–132.0 µM). This effect on PBMCs is fascinating and may suggest that B18 may affect the behavior of PBMCs in various activation states.

Cancer therapeutic peptides are promising tools for the treatment of various cancers, and this class of drugs has several important advantages over other biologics, such as, proteins or antibodies. Peptides are small, and thus easy to synthesize and to permeate the cell membranes. In addition to their small size, peptides can be derived from natural proteins, and they can be engineered to maintain the unique structural and functional properties of the natural protein [68,69]. Peptides can also be modified easily [70] and are less immunogenic compared to proteins and antibodies [71]. Above all, the specificity and affinity of peptides to their targets have the potential to minimize drug-drug interactions and toxic side effects. Like all peptides, B18 appears to have a short half-life, which may affect its stability and bioavailability *in vivo*. Despite this drawback, the B18-mediated blockade of cancer cell adhesion is a property that may be harnessed in future drug development efforts. Indeed, a number of anti-adhesion peptides including arginine-glycine-aspartate (RGD), derived from the common conserved sequences of fibronectin, collagen, and fibrinogen [72]; YIGSR, derived from the basement membrane protein laminin [72], and EILDV, derived from the core sequence of fibronectin [73] have been described. Thus, the anti-adhesion property of B18 has significance for understanding cross-talking pathways of cell-cell or cell-ECM adhesion for the purpose of interrupting adhesion-dependent biological events that promote breast cancer. The atomistic and functional properties of B18 described in this manuscript shade light on the potential mechanism of action of B18 and forms a basis for i) developing B18 as a compound that blocks adhesion-dependent oncogenic events, and ii) designing highly potent B18-derived membrane-active peptides that may be capable of modulating membrane properties. 

## 4. Materials and Methods 

Ethics: Blood samples for isolation of PBMCs were collected from study participants who at the time of collection, had no symptoms of infection and reported not using illicit substances. Studies were conducted according to University regulations approved by Stony Brook University Institutional Review Boards (IRB # 201900507).

Chemical reagents: Roswell Park Memorial Institute (RPMI) 1640 media, Dulbecco’s Modified Eagle Medium (DMEM) media, penicillin-streptomycin, Amphotericin B, l-gutamine and 1x Dulbecco’s phosphate buffered saline (DPBS) were obtained from Corning, Thermofisher, Grand Island, NY, USA. 4-(2-hydroxyethyl)-1-piperazineethanesulfonic acid (HEPES) was obtained from Fisher Biotech, Fair Lawn, NJ, USA. Fetal bovine serum (FBS) was obtained from Atlanta Biologicals, Flowery Branch, GA, USA. Ficoll-Paque PLUS was obtained from Amersham Biosciences, Uppsala, Sweden. Tris was from Thermos Scientific, Fair lawn, NJ, USA. Fibronectin and collagen type I were obtained from Corning, Bedford, MA, USA. 3-(4,5-dimethylthiazol-2-yl)-2,5-diphenyl tetrazolium bromide (MTT) reagent, 1,4-Dithiothreitol (DTT), Iodoacetic acid (IAA), PKH67 Green Fluorescent Cell Linker Kit for General Cell Membrane Labeling, and chloroform were purchased from Sigma-Aldrich, St. Louis, MO, USA. Trypsin was obtained from Worthington Biochemical Corporation, Lakewood, NJ, USA. Acetic acid was obtained from Macron Fine Chemicals, Radnor, PA, USA. 1-palmitoyl-2-oleoyl-glycero-3-phosphocholine (POPC) was obtained from Avanti polar lipids, Alabaster, AL, USA. 

Cell Lines: MDA-MB-468, MDA-MB-231, ZR-75-1, MCF7, and T47D were obtained from the Physical Sciences-Oncology Network (PS-ON) Bioresource Core Facility (PBCF) at American Type Culture Collection (ATCC, Manassas, VA, USA). SKBR3 cell line was a kind gift from Dr. Hyungjin Kim at Stony Brook University. ZR-75-1, MCF7, and T47D were maintained in complete RPMI 1640 media. MDA-MB-468, MDA-MB-231, and SKBR3 were maintained in complete DMEM media. Both complete RPMI and DMEM were supplemented with 10% FBS, 1% penicillin-streptomycin, 1 µg/mL Amphotericin B, 1% of l-glutamine, 2 mM sodium pyruvate, and 10 mM HEPES buffer at pH 8. Human Peripheral Blood Mononuclear Cells (PBMCs) were isolated from fresh human blood by Ficoll-Paque PLUS and cultured in complete RPMI 1640 media.

Peptide synthesis: As described previously [32], B49Mod1 is an analog of B49 with two protection residues on each terminus. B18 is the last 18 residues of B49Mod1. Both B49Mod1 and B18 were synthesized by ChinaPeptides (Shanghai, China) with a purity of >95%. The peptides were reconstituted following the manufacturer’s instruction. Briefly, 0.02% acetic acid in ultra-pure water was used to reconstitute the peptide into 5 mg/ml aliquots and stored at -20° C until used for the other experiments. 

Circular Dichroism (CD) analysis: POPC liposome was prepared as described previously [74,75]. Briefly, POPC was dissolved in chloroform and allowed to dry, leaving a thin lipid film in a glass vial. The film was then hydrated with Tris buffer (0.5 mM, pH7.4) to generate multilamellar vesicles (LUVs) at room temperature. The LUVs suspension was extruded through 1000 nm polycarbonate filters to obtain the small unilamellar vesicles (SUVs). CD analysis of the peptides (B49Mod1 and B18) with or without POPC SUVs was collected through a Jasco J-715 spectrophotometer (Jasco Analytical Instruments, Easton MD). A variety of peptide/lipid ratios were prepared and applied to the measurement. The full-spectrum collection was taken from 190 to 260 nm at 0.1 nm resolution with a scan rate of 100 nm/min. Each sample was measured three times with a 1 mm path length quartz cuvette. The data were analyzed by the CAPITO tool [76]. 

Cell adhesion to collagen or fibronectin: Adhesion assays were conducted as previously described [3,4,5]. Briefly, 96-well plate was coated with fibronectin or collagen type I at 37 °C for 2 hours. The incoming cells pre-treated with peptides (0.25 mg/ml), or an equivalent volume of vehicle (control) for 2 hours before PKH67Green fluorescent cell linker labeling were added atop collagen- or fibronectin-coated wells. Cells were cultured for 4 or 8 hours, after which non-adhered cells were removed by washing with 1x DPBS. Cell adhesion was determined by measuring fluorescence intensity using the BioTek Synergy H1 microplate reader (BioTek, Winooski, VT, USA). 

Cell to cell adhesion: 96-well plates were seeded with 4T1 shCTL- or shBST-2-expressing cells, cultured for 24h, and confluent monolayers formed [3,4,5]. Incoming cells were labeled with PKH67Green fluorescent cell linker. Twenty-thousand cells/well were added to the monolayers, and cells were allowed to incubate for 4 hours. Incoming cells that did not adhere were washed off with 1x DPBS and plates were read using a Tecan Infinite M200 Pro plate reader (Tecan Group Ltd.; Mannedorf, Switzerland) at 485 nm/535 nm (excitation/emission) wavelength to assess the degree of cell adhesion. Values for adhesion assays were plotted as relative fluorescence intensity (RFI). The variation of RFI values from experiment to experiment correspond to deferent uptake rates of PKH67 green dye by cells. For adhesion assays assessing the activity of B49 or B49Mod1, the aforementioned protocol was used except that cell monolayers were treated with peptide for 4 hours and then washed once with 1x DPBS before the addition of PKH67Green-labeled cells.

Viability Assay: Equivalent numbers of cells from each cell line was seeded in 96-well plate and incubated at 37 °C for 24 hours before treatment. The cells were treated with different concentration of peptides and incubated for 24 hours at 37 °C. 20 µl of 5mg/ml of MTT reagent was added to each well and incubated for 4 hours at 37 °C. The supernatant was then removed, followed by addition of 150 µl of MTT solvent, with shaking 15 min at room temperature. Absorbance at 590 nm was then measured through the BioTek Synergy H1 microplate reader.

Deactivation of B49Mod1 by DTT and IAA: To inhibit the ability of B49Mod1 to form cysteine-mediated disulfide bonds, 20 µg of B49Mod1 was treated with 2 µg of the reducing agent DTT for 30 minutes at 56 °C. Samples were either frozen at −20 °C or treated with 5 µl of the alkylating agent IAA at 5 mg/ml at room temperature for 30 minutes to prevent reformation of disulfide bonds. To quench unreacted IAA, an extra 2 µg of DTT was added to the reaction, and samples were incubated for 15 minutes at room temperature in the dark. All reactions were used in the adhesion assay.

Digestion of B49Mod1 with trypsin: 20 µg of B49Mod1 was treated with 0.2 µg of Trypsin for 16 hours at 37° C with 5% CO_2_. After incubation, samples were frozen at −20° C until used for chromatogram analyses. B49Mod1 degradation by serum and cellular proteases: 20 µg of B49Mod1 was treated with 0, 5, 10 or 100% FBS in RPMI for 16 hours at 37° C with 5% CO_2_. Following incubation, samples were frozen at −20° C until used for adhesion assays. In addition, conditioned media (originally 5% FBS in RPMI) was collected from MDA-MB-231 cells after 24 hours of culture and 60 µl of the media were added to 20 µg of B49Mod1 and incubated for 16 hours at 37° C with 5% CO_2_ to assess the stability of B49Mod1. Following incubation, samples were stored at −20° C until used for adhesion assays. 

Molecular Dynamics (MD) Simulation: The system set up for MD simulations was previously described [59,61,77]. Briefly, the initial structures of two different systems for the MD simulations were constructed using the CHARMM-GUI tool [43,78]. For the peptide•BST-2 simulation, peptide chains a and b were placed at the same distance from the center of mass (COM) of BST-2. For peptide•membrane simulation, a single peptide was initially placed either atop or inside the lipid bilayer (peptide/lipid ratio = 1/128), or a bundle of four peptides was initially placed inside the lipid bilayer (peptide/lipid ratio = 4/128) with most of the hydrophilic residues facing inwards. All systems were simulated using the TIP3P water model in the presence of 0.15 M KCl, and the final system was neutral in charge [61]. The simulation input was prepared under periodic boundary conditions (PBC) with the Particle mesh Ewald (PME) method [49]. The GROMACS/5.1.1 [54] software and CHARMM36 force field [43,79] were used through the simulation. For each simulation system, minimization was performed before slowly heating the system to 303 K, which is above the phase transition temperature for both POPC and POPS [80] and stopped when the maximum force was less than 1000 kJ mol^−1^ nm^−1^. The simulation was carried out in the constant volume (NVT) ensemble until the target temperature (303K) was reached. A constant pressure (NPT) ensemble was applied for the following simulation up to 1000 ns with a time step of 2 fs. The trajectory was analyzed through VMD 1.9.3 [81] and GROMACS.

Free energy calculations: Potential of mean force was calculated through umbrella sampling MD simulation. The system was setup as previously described [66,67]. Briefly, pulling simulations were first performed to generate the initial structure for the umbrella sampling simulation. The center of mass (COM) of the peptide was pulling into the COM of the lipid membrane along the z-direction with the constant velocity and force constant set as 0.00001 nm ps^−1^ and 1000 kJ mol^−1^ nm^−2^, respectively. Thirty-six frames were selected from the pulling simulation trajectories and used as the initial structure for each window of the umbrella sampling simulation, while the window interval was 0.1 nm. Each window system was simulated with minimization, equilibration, and production steps. All ensembles, temperature, and pressure were maintained as described in the previous section. Each window was subjected to production simulation for 200 ns, and the last 50 ns was split into five blocks. The error bars represent the standard error of the blocks’ average. The free energy profile was calculated using the weighted histogram analysis method (WHAM).

Statistics: Statistical analysis of significant differences were performed with ordinary one-way ANOVA test (Dunnett’s correction) and Two-tailed t test (Welch’s correction) using GraphPad Prism software, San Diego, CA, USA. *p = < 0.05, **p = < 0.01, ***p = < 0.001, ****p = < 0.0001 and ns = non-significant. Error bars represent standard error of the mean (S.E.M.). 

## Figures and Tables

**Table 1:** Sequence information and characteristics of B49Mod1 predicted fragments.

ID	Sequence	Number of Cysteine	Charge	H (kcal/mol)	µH (kcal/mol)	MW (kDa)
B3	TIK	0	1	0.357	0.762	0.360
B7	ANSEACR	1	0	−0.167	0.248	0.750
B4	DGLR	0	0	−0.020	0.722	0.460
B6	AVMECR	1	0	0.442	0.295	0.708
B15	NVTHLLQQELTEAQK	0	−1	0.250	0.360	1.752
B18	GFQDVEAQAATCNHTVMA	1	−2	0.358	0.222	1.893

H = Hydrophobicity, µH = Hydrophobic moment, and MW = Molecular weight.

## Supplementary Materials

The following are available online at https://www.mdpi.com/1420-3049/25/5/1188/s1. Figure S1: Number of hydrogen bonds between each residue of B18 chain a with BST-2 protein. Figure S2: Structural properties of B18 chains inside the 1-palmitoyl-2-oleoyl-glycero-3-phosphocholine (POPC) membrane. (A) Snapshots of each B18 chain at 0, 250, 500, 750, and 1000 ns. (B) Root-mean-square deviation (RMSD) of each B18 chains as a function of time. (C) The helicity of each B18 chain as a function of time. Figure S3: Formation of hydrogen bonds by B18 peptides inside the POPC membrane. (A) The number of H bonds between different B18 peptide chains. (B) Representative images of hydrogen bond between B18 peptide chains. (C) The number of hydrogen bonds between each B18 chain with POPC (left) or H2O (right) as a function of time.
