# Supplementary material for: Development and Characterization of the Shortest Anti-Adhesion Peptide Analogue of B49Mod1
Source: Molecules. 2020 Mar 6;25(5):1188. doi: 10.3390/molecules25051188 (PMC7179399; doi:10.3390/molecules25051188)
Supplement: Supplementary file 1 [file molecules-25-01188-s001.pdf]

Supplementary Materials: The following are available online at www.mdpi.com/xxx/s1.

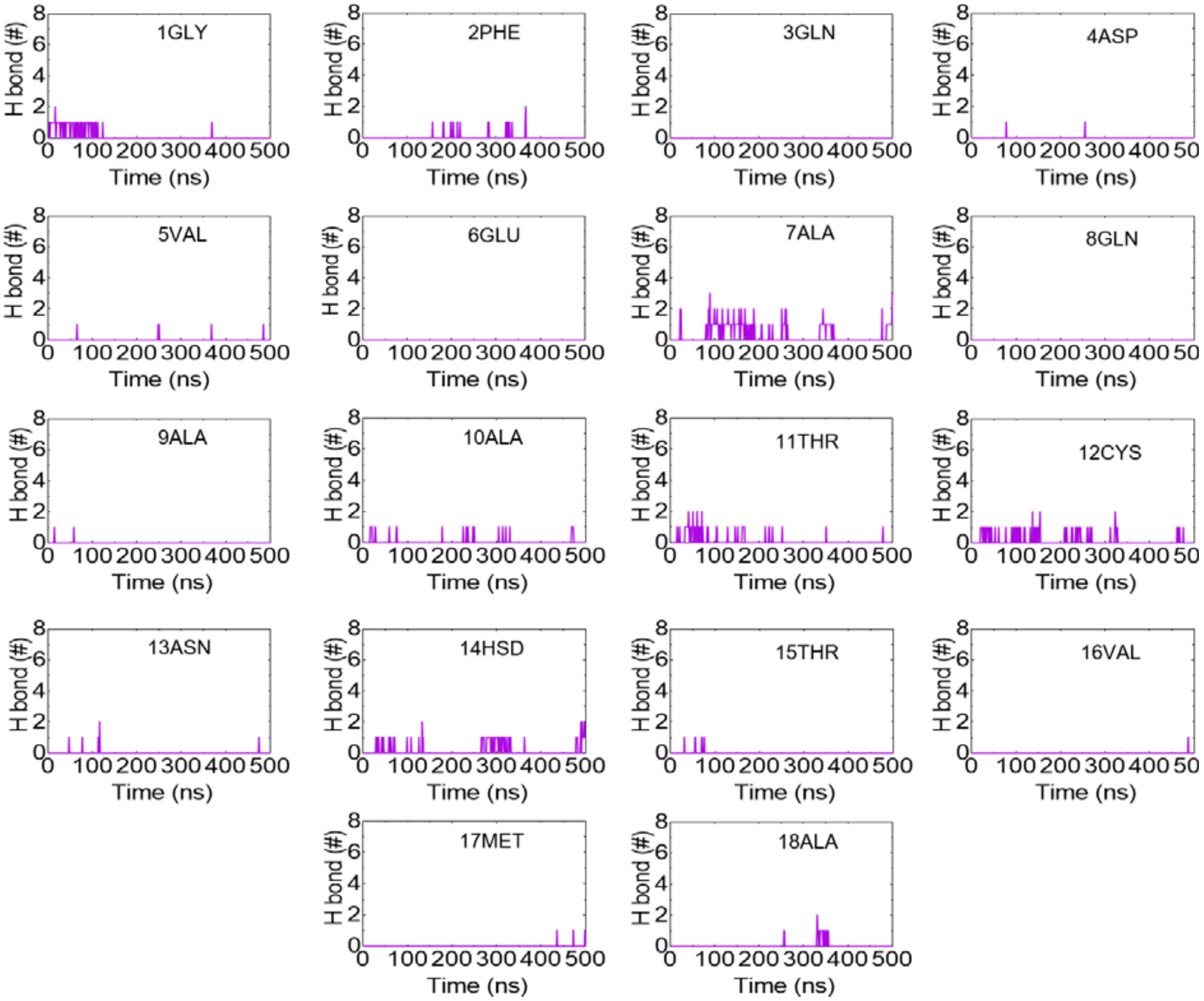

Figure S1: Number of hydrogen bonds between each residue of B18 chain a with BST-2 protein.

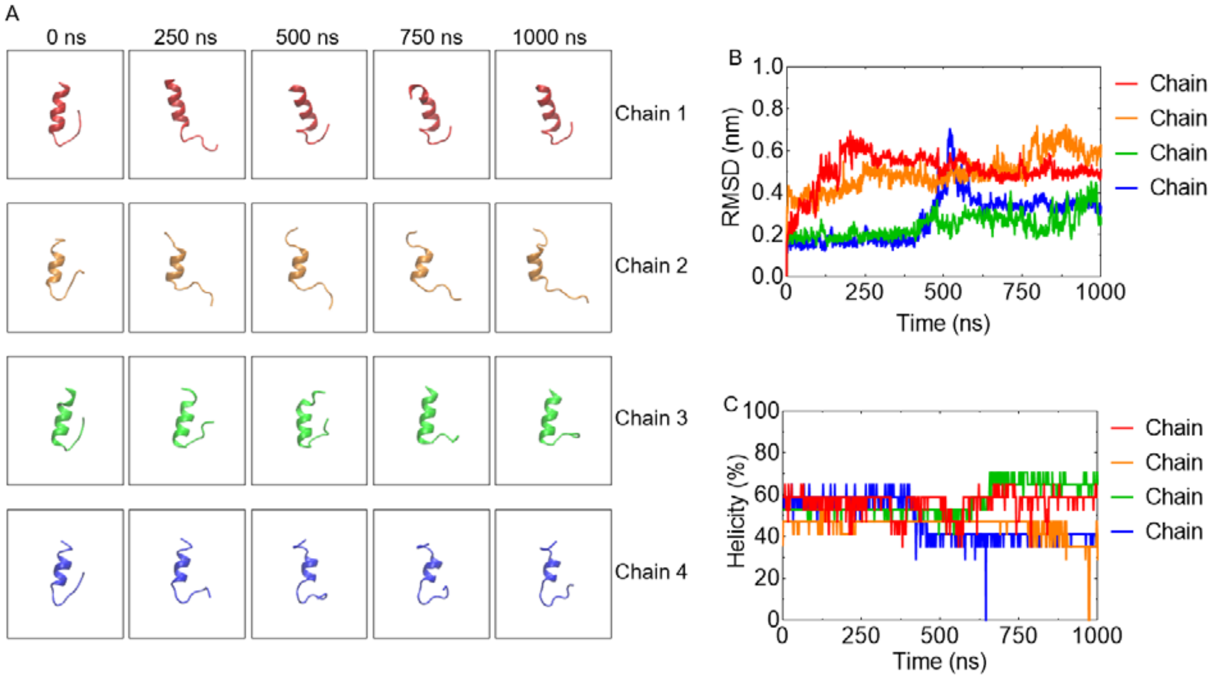

Figure S2: Structural properties of B18 chains inside POPC membrane. (A) Snapshots of each B18 chain at 0, 250, 500, 750 and 1000 ns. (B) RMSD of each B18 chains as a function of time. (C) Helicity of each B18 chains as a function of time.

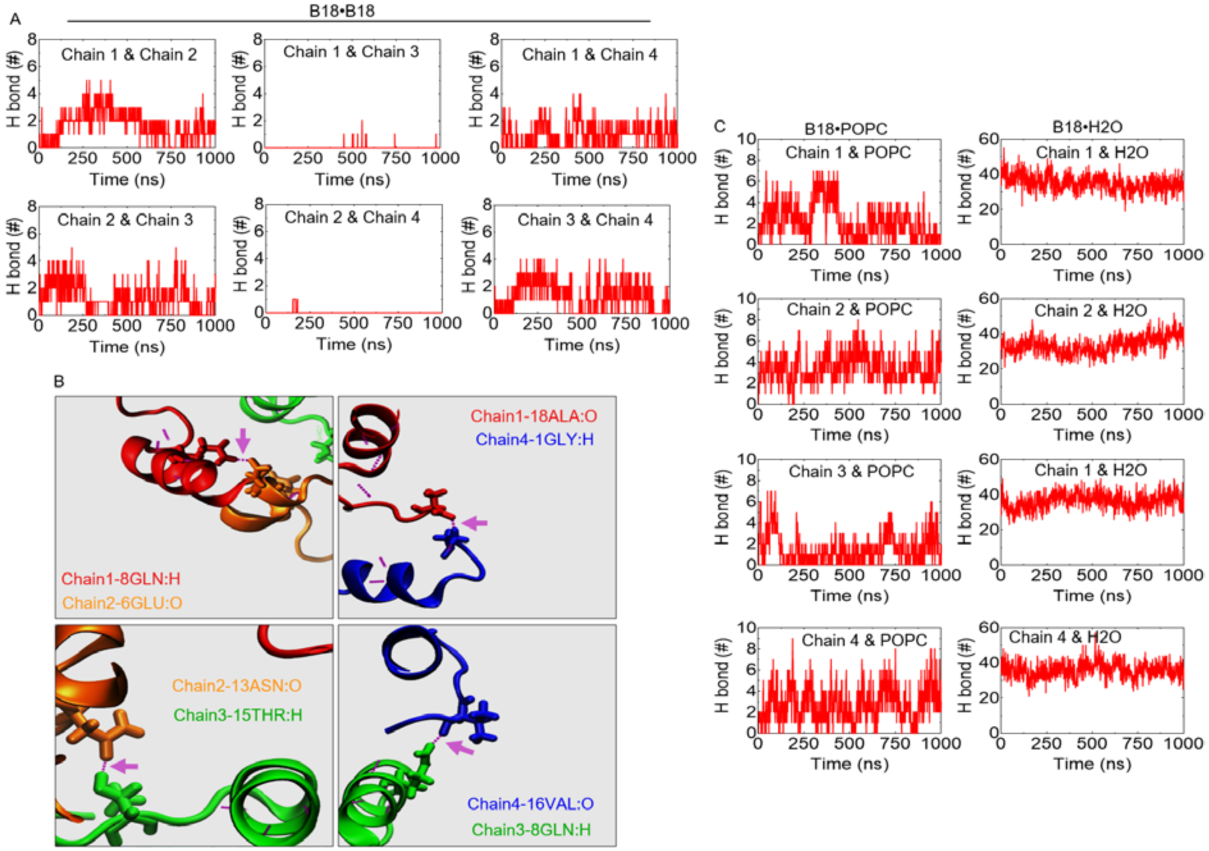

Figure S3: Formation of hydrogen bonds by B18 peptides inside POPC membrane. (A) Number of H bonds between different B18 peptide chains. (B) Representative images of hydrogen bond between B18 peptide chains. (C) Number of hydrogen bonds between each B18 chains with POPC (left) or H2O (right) as a function of time.
